# Direct sequencing of human gut virome fractions obtained by flow cytometry

**DOI:** 10.3389/fmicb.2015.00955

**Published:** 2015-09-08

**Authors:** Mária Džunková, Giuseppe D’Auria, Andrés Moya

**Affiliations:** ^1^Área de Genómica y Salud, Fundación para el Fomento de la Investigación Sanitaria y Biomédica de la Comunidad Valenciana – Salud Pública, ValenciaSpain; ^2^Instituto Cavanilles de Biodiversidad y Biología Evolutiva, Universitat de València, ValenciaSpain; ^3^CIBER en Epidemiología y Salud Pública, MadridSpain

**Keywords:** human gut virome, fluorescent activated cell sorting, *de novo* assembly, whole genome amplification, bacteriophages

## Abstract

The sequence assembly of the human gut virome encounters several difficulties. A high proportion of human and bacterial matches is detected in purified viral samples. Viral DNA extraction results in a low DNA concentration, which does not reach the minimal limit required for sequencing library preparation. Therefore, the viromes are usually enriched by whole genome amplification (WGA), which is, however, prone to the development of chimeras and amplification bias. In addition, as there is a very wide diversity of gut viral species, very extensive sequencing efforts must be made for the assembling of whole viral genomes. We present an approach to improve human gut virome assembly by employing a more precise preparation of a viral sample before sequencing. Particles present in a virome previously filtered through 0.2 μm pores were further divided into groups in accordance with their size and DNA content by fluorescence activated cell sorting (FACS). One selected viral fraction was sequenced excluding the WGA step, so that unbiased sequences with high reliability were obtained. The DNA extracted from the 314 viral particles of the selected fraction was assembled into 34 contigs longer than 1,000 bp. This represents an increase to the number of assembled long contigs per sequenced Gb in comparison with other studies where non-fractioned viromes are sequenced. Seven of these contigs contained open reading frames (ORFs) with explicit matches to proteins related to bacteriophages. The remaining contigs also possessed uncharacterized ORFs with bacteriophage-related domains. When the particles that are present in the filtered viromes are sorted into smaller groups by FACS, large pieces of viral genomes can be recovered easily. This approach has several advantages over the conventional sequencing of non-fractioned viromes: non-viral contamination is reduced and the sequencing efforts required for viral assembly are minimized.

## Introduction

Traditional methods of virus discovery are based on the employment of electron microscopy, cultivation, and PCR amplification. In recent years, high-throughput shotgun sequencing opened up new possibilities for viral diversity exploration, and it has already been applied to complex environments such as the human body (Kristensen et al., 2010; Ladner et al., 2014).

However, the high-throughput shotgun sequencing of viromes encounters several difficulties. Despite rigorous efforts to eliminate non-viral particles in environmental samples by filtering and centrifugation processes (Thurber et al., 2009), the majority of assigned sequences still match bacteria and eukaryotic DNA, which usually form up to 70% of the total assigned DNA sequences (Breitbart et al., 2003; Reyes et al., 2010). Moreover, due to the fact that the sequence databases are still incomplete or biased toward the most studied human viruses, up to 80% of the reads of viral metagenomes yield no significant matches against public sequence databases (Edwards and Rohwer, 2005; Vázquez-Castellanos et al., 2014). Thus, only if enormous sequencing efforts are applied can the assembly of unassigned reads be achieved and novel organisms be described (Dutilh et al., 2014).

Another limitation in virus discovery is that the high-throughput sequencing platforms require 10s of nanograms or even micrograms of DNA for sequencing library preparation, however, the DNA yields in virus extractions are usually below 1 ng (Duhaime et al., 2012). This requirement resulted in an increasing trend to amplify extracted DNA before sequencing, e.g., by non-specific PCR amplification of DNA fragments (Stang et al., 2005; Ambrose and Clewley, 2006; Solonenko et al., 2013) or whole genome amplification (WGA) (Breitbart et al., 2008; Pérez-Brocal et al., 2013; García-López et al., 2014). However, DNA enrichment methods are prone to developing chimeras, GC-content biases, and non-target DNA contaminations (Pinard et al., 2006; Lasken and Stockwell, 2007). Moreover, WGA can also lead to an over-amplification of certain virus types (Kim et al., 2008). Nevertheless, Džunková et al. (2014) demonstrated that if the concentration of extracted DNA does not reach the minimal limit required by the sequencing library preparation protocols, the sample can still be successfully sequenced if the concentration of the completed library contained the number of molecules required for loading on the sequencing plate.

In this work we propose a method to improve viral sample preparation prior to sequencing, in turn enhancing virome sequence assembly. For more precise detection of viral particles, we employed fluorescent-activated cell sorting (FACS). While previous studies used FACS with seawater viral samples or already known cultivated phages (Bettarel et al., 2000; Chen et al., 2001; Allen et al., 2011), in this work we attempt to apply FACS for the sorting of viral particles from an extremely complex environment such as human fecal samples, starting from quantities of DNA below the detection limit of PicoGreen assay (less than 0.25 pg/μl). Therefore, we optimized the protocol avoiding the WGA and sequencing using the DNA extracted directly from the separated viral sample, which contained a few 100 viral particles.

## Materials and Methods

### Preparation of Viral Sample from Human Feces

A healthy volunteer provided the fecal sample used in this study. The institutional review board approved the study, and informed consent was obtained.

**FIGURE 1:**
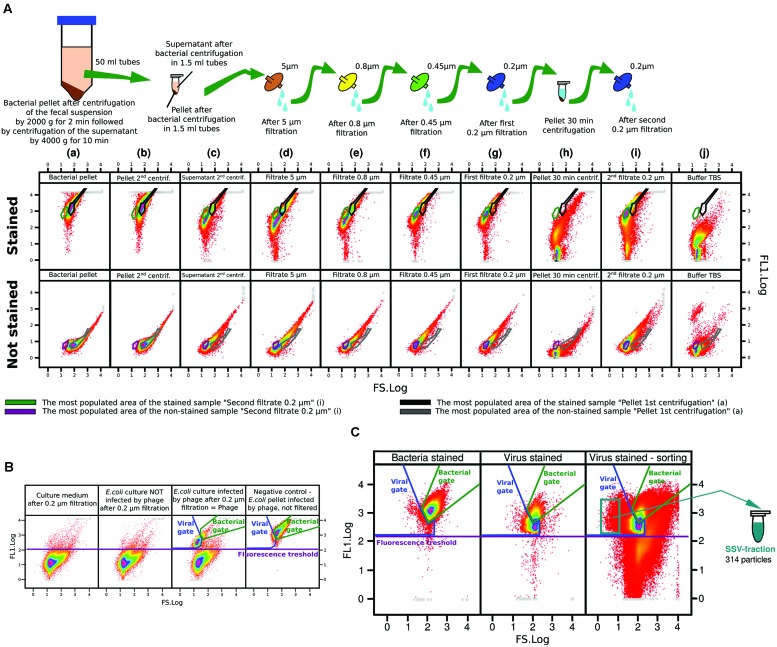
**Flow cytometry bi-plots of all control samples and the fecal viral sample. (A)** FC bi-plots of stained and unstained control samples across all steps of the fecal purification protocol. **(B)** FC bi-plots of *Escherichia coli* culture infected/not infected by phage M13KE. **(C)** FACS of the fecal sample.

The workflow of the viral sample preparation protocol present in this study is graphically visualized in Supplementary Figure S1. Purification of the viral fraction started with 30 ml of fecal sample, which was divided into equal parts of six 50 ml tubes and resuspended in 30 ml of sterile TBS (Tris-buffered saline, composed of 50 mM Tris, 150 mM NaCl, sterilized by autoclaving and filtered through 0.2 μm pore filters). The fecal suspension was purified by subsequent centrifugation and the filtration steps described below. During each step, control samples were taken for sample purity control by FC, shown in **Figure 1A**, where the samples were either stained by SYBR Green I or left unstained.

First, the fecal suspensions were centrifuged at 2,000 *g* for 2 min at 4°C. In this stage the supernatants contained both bacteria and viruses, while the pellet was formed by large stool particles. The supernatants were centrifuged twice at 4,000 *g* for 10 min at 4°C (Eppendorf 5810 R centrifuge): the pellet contained bacteria and the supernatant contained viral particles with some remaining bacteria, which will be removed later (**Figure 1A**a). Ten microliter of the formed bacterial pellet was transferred to a fresh 50 ml tube and resuspended in 16 ml of fresh TBS representing a FACS control sample containing only bacterial pellet. The supernatants were collected and distributed into 1.5 ml tubes and centrifuged at 16,000 *g* for 45 min at 4°C (Eppendorf 5415 R centrifuge). As shown in **Figure 1A**b,c, some of the remaining bacteria was collected by centrifugation, but some bacteria were still present in the supernatant. The supernatant of all 1.5 ml tubes were pooled and filtered consecutively per 5, 0.8, 0.45, and 0.2 μm filters. The particles present in the sample after each filtration step is shown in **Figure 1A**d–g.

To ensure that the last filtrate does not contain any non-viral particles, it was distributed into 1.5 ml tubes and centrifuged at 16,000 *g* for 30 min at 4°C. The FC visualization of the stained pelleted particles showed that they did not reach the fluorescence threshold of particles containing DNA set according to the filtered TBS (**Figure 1A**h,j). It indicated that the obtained pellet was formed by complex organic molecules present in feces, but not by DNA viruses. To ensure that the supernatant was really bacteria-free, the filtration through 0.2 μm pores was repeated with the pooled supernatant of all the 1.5 ml tubes (cca 16 ml) (shown in **Figure 1A**i).

In order to digest any unencapsulated DNA or RNA of non-viral origin, a nucleases mixture containing 14 U Turbo DNAse (Life Technologies, Ref. AM2238), 20 U bensonase (Novagen, Ref. 70746-4), and 20 U RNAse A (Roche, Ref. 10109142001) was added to each sample and incubated for 1 h at 37°C, and then inactivated by incubation at 75°C for 15 min (as described by Pérez-Brocal et al., 2013). Viral particles were then concentrated adding 4 ml of 2.5 M NaCl/20% PEG-8000 (PEG-NaCl) to the filtered supernatant. For the FC control of bacterial pellet only, the same volume of PEG-NaCl was also added to bacterial pellet resuspended in 16 ml of TBS, which was saved after bacterial centrifugation at the beginning of the protocol (described above). The tubes were vortexed, stored on ice for 1 h and, after precipitation, were centrifuged at 4,000 *g* for 30 min. After this step the samples contained concentrated viral particles, while the bacterial control sample taken after the first centrifugation contained concentrated bacteria also coated by PEG-NaCl.

The concentrated pellet of particles were then resuspended in 900 μl of TBS and fixed with 100 μl of 37% formaldehyde and incubated for 1 h at 4°C. After fixation, the samples were purified with TBS: 200 μl of PEG-NaCl was added to each sample, incubated on ice for 30 min and later centrifuged at 16,000 *g* for 30 min. The supernatant was discarded and the pellet was resuspended in 90 μl TBS. Ten μl of 10x diluted SYBR Green I nucleic stain (Life Technologies, Ref. S-7563) was added to five of the tubes containing viruses and to the bacterial pellet, heated at 80°C for 10 min and left to cool down, similar to that described by Brussaard (2004), while the sixth tube was left unstained as a negative control. The volume of samples was brought to 1 ml with the filtered TBS, as required by the specifications of the flow cytometry equipment. We proceeded with cell sorting within a 2 h timeframe.

### Preparation of Phage Control

Three flasks with 100 ml of LB (lysogeny broth) medium were prepared. The first flask was inoculated with 200 μl of overnight culture of *Escherichia coli* ER2738 (New England Biolabs, Ref. E4104S); the second flask with the same overnight culture and with 1 μl of M13KE Phage (New England Biolabs, Ref. N0316S); the third flask was not inoculated and left as a negative control without any bacteria. The three flasks were left to grow for 4 h at 37 °C with shaking at 250 rpm. The culture was transferred into 50 ml tubes and processed using the same protocol described above for the fecal samples, keeping 10 μl of the bacterial pellet (*E. coli* and *E. coli* infected by phage) as a control, similar to the case of fecal sample explained above.

### Flow Cytometry of Viral Samples

Fluorescence activated cell sortings was carried out using the MoFlo XDP Cell Sorter (Beckman Coulter, Ref. ML99030). The light sources were from the Argon 488 nm (blue) laser (200 mW power) and the 635 nm (red) diode laser (250 mW power). The lasers were aligned using 10 μm Flow-Check beads (Beckman Coulter, Ref. 6605359) and 3 μm beads Flow Set (Beckman Coulter, Ref.6607007). The cytometer emission filter used was the 520/30 (FL1) in order to obtain the SYBR Green I emission. The trigger was set on side-scatter. The first FC bi-plot has been set on side scatter vs. forward scatter and particles with equal or smaller sizes than bacteria have been preselected. The preselected FC events have been visualized on a second bi-plot showing forward scatter vs. fluorescence (channel FL1), which has been used for cell sorting. The samples containing stained bacterial pellet were used as a control to localize the events, with the size of bacterial cells and phage M13KE control being used for the detection of the area corresponding to the viruses (**Figures 1A,B**). The particles from the selected fraction were sorted into sterile Lo Bind 1.5 ml tubes (Eppendorf, Ref. 0030 108.051).

### DNA Extraction and Sequencing

**FIGURE 2:**
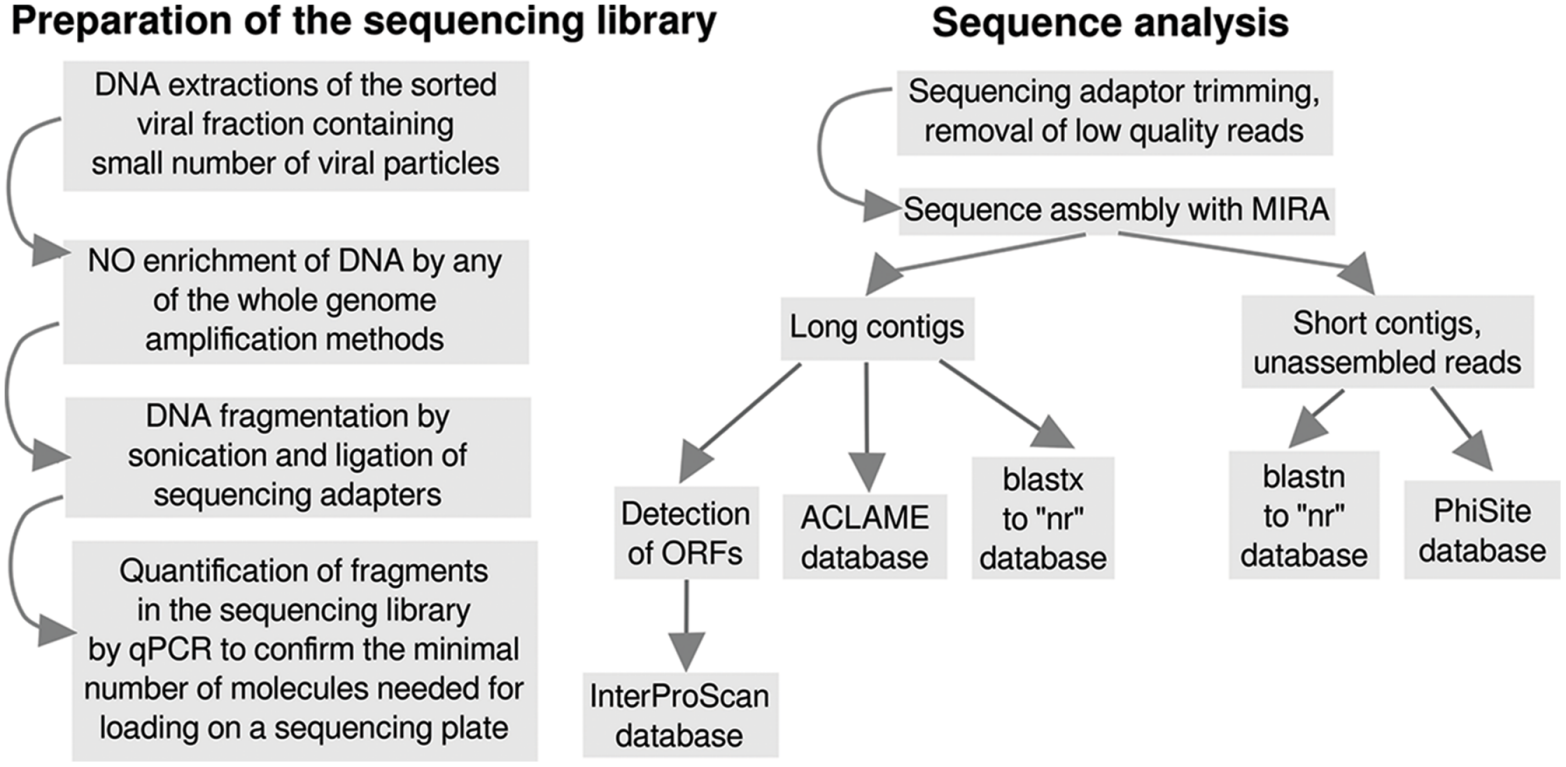
**Workflow diagrams of the sequencing library preparation protocol and of the data analysis**.

The DNA was extracted according to the protocol set by Ausubel et al. (1992) in sterile conditions. All the chemicals used were previously sterilized using an autoclave and filtered through 0.2 μm sterile filters. For the shotgun library preparation, the manufacture’s (Roche Applied Science) standard protocol was replaced with the optimized protocol for limited DNA samples (Džunková et al., 2014). The exact number of molecules present in the 454 shotgun library concentration was determined by qPCR by probes specific for custom “Y” 454 library adaptors, as described by Zheng et al. (2011). After the quantification step, the emPCR and sequencing with a GS FLX Titanium Sequencing XLR70 Kit (Roche Applied Science, Ref. 5233526001) were carried out following the standard protocols on 1/8 of the 454 picotiterplate. The workflow of the sequencing library preparation is shown in **Figure 2**.

### Data Analysis

The obtained sequences were filtered, quality trimmed, and adaptors were removed using Roche’s SFFINFO tool and then they were double-checked for the presence of Y adaptors in the 3′ end DNA using Biostrings v.2.11 package (Pages et al., 2014) in R programming language (R Development Core Team, 2008). Low complexity reads (entropy <50), low quality reads (<25), short reads (<50 bp), and erroneous reads (>5% N bases) were removed using PRINSEQ (Schmieder and Edwards, 2011). Sequences were assembled with MIRA3 (Chevreux et al., 1999) using *de novo* genome accurate 454 settings, permitting the assembly of as few as two reads per contig.

The open reading frames (ORFs) in the larger contigs (>1,000 bp) were identified by Glimmer3 (Delcher et al., 1999) and annotated by an InterProScan search using all available databases, combining the individual strengths of these different annotation sources and providing comprehensive information about protein families, domains, and functional sites (Quevillon et al., 2005). The maps of ORFs detected in the contigs was constructed using the genoPlotR package (Guy et al., 2010) in R programming language. Moreover, the larger contigs (>1,000 bp) were annotated using the “blastx” algorithm using the “nr” database (Altschul et al., 1990). To decide whether a sequence could be classified as virus/phage by “blastx”, we used an approach previously used by Law et al. (2013), revising the 100 best matches. Moreover, the same contigs were analyzed by searching on the ACLAME database of mobile genetic elements (Leplae et al., 2004).

Contigs shorter than 1,000 bp and unassembled reads were annotated by “blastn” on the “nr” database using the “megablast” algorithm (Altschul et al., 1990). The taxonomy assignation of the best GI matches were retrieved by a script written in R programming language using the ape package (Paradis et al., 2004). The frequency of all genera, as well as the ten most frequent genera across the two datasets were compared by Pearson correlations. These sequences were also compared with the phiSITE database containing only viral genomes (Klucar et al., 2010).

The workflow of the data analysis is shown in **Figure 2**.

### Data Access

Sequences were deposited on the EMBL-EBI Sequence Read Archive (SRA) with the study number PRJEB7515.

## Results

### FACS and Sequencing Results

The particles derived from the selected FACS area corresponded to the viral particles of the smallest size. The sorted fraction was labeled SSV-fraction (small size viruses). The density of events in this area was very low: 314 particles of SSV-fraction (0.016%) were sorted out of a total of 1,904,265 particles. The gate for the selected SSV-fraction is marked in **Figure 1C** in a green–blue color.

**FIGURE 3:**
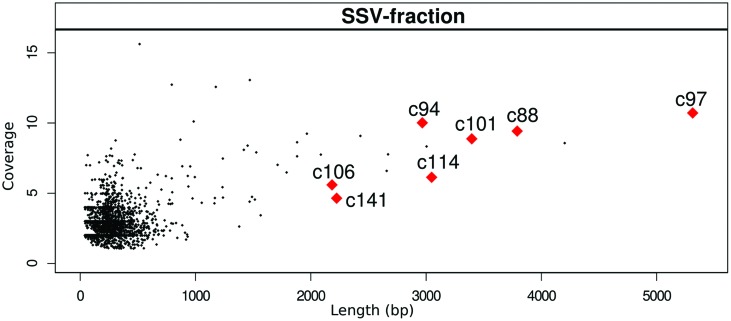
**Length and coverage of all assembled contigs in SSV-fraction**. Larger red spots mark the contigs that had explicit matches to phage-related proteins in the three approaches used in our study (“blastx”, ACLAME and InterProScan).

The reads of SSV-fraction generated 17.07 Mbp (60,030 reads with a mean length of 283.49 bp) and were assembled into 2,475 contigs; 34 of them were longer than 1,000 bp with 15.26× average coverage (maximum coverage 27×). The largest contig was of 5,313 bp (**Figure 3**).

### Analysis of Long Contigs

**FIGURE 4:**
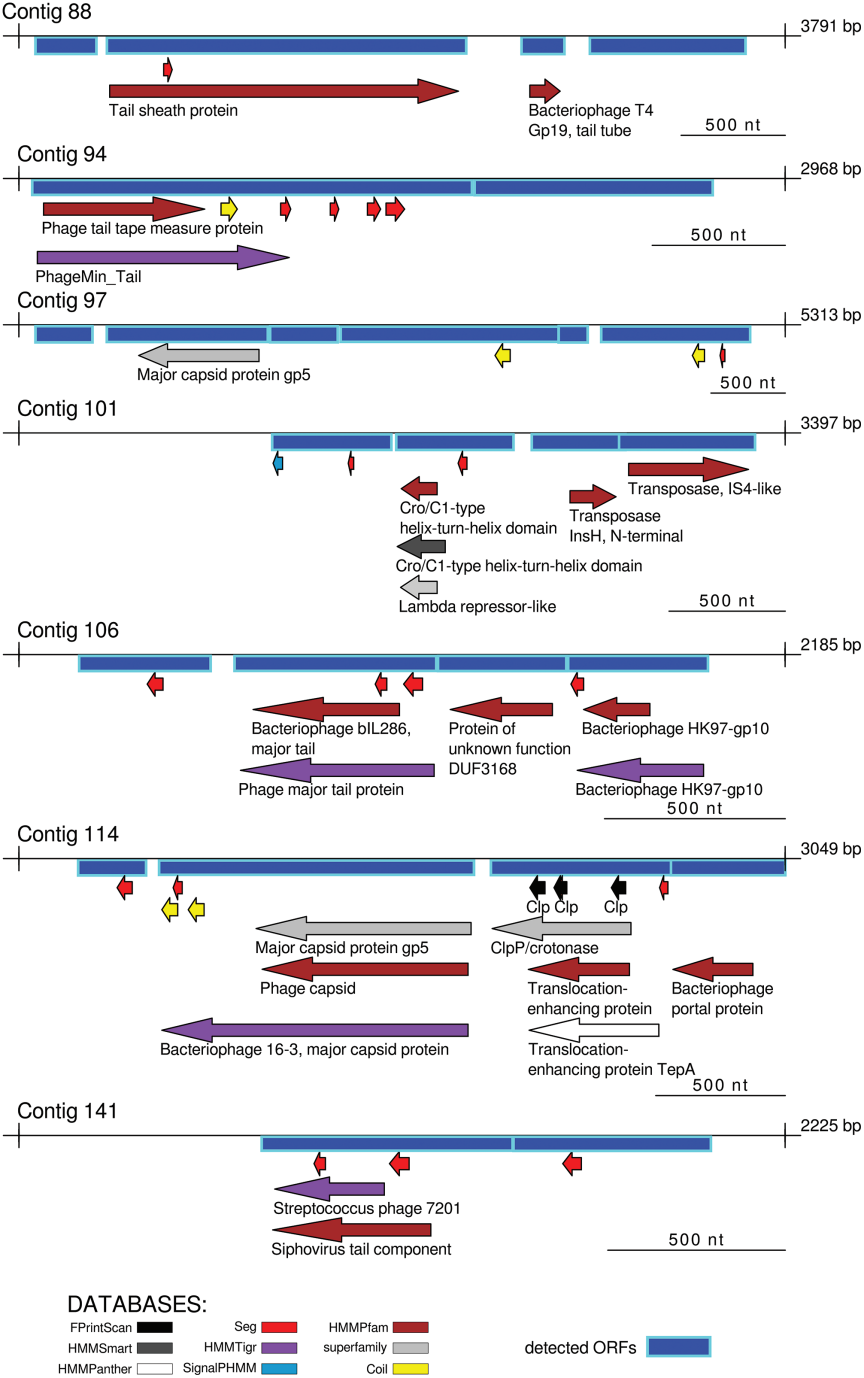
**Visualization of contigs with explicit matches to phage-related proteins by InterProScan.** Annotation by all searching tools included on InterPro database is shown, as well as ORFs detected by Glimmer. The remaining contigs with no explicit matches to phage-related proteins by InterProScan are shown in Supplementary Figure S2.

In total, 89 ORFs were detected in the 34 long contigs. ORFs encoding sequences of bacteriophage-related proteins were detected in seven of them, such as bacteriophage capsid proteins, bacteriophage tail proteins, virus tail components, translocation-enhancing proteins, lambda phages transposase, and gene transfer agent portal proteins. Twenty-four contigs contained only protein prediction sites, such as coiled coils (Lupas et al., 1991) or low complexity regions “seg” and four contigs remained unannotated by InterProScan. The annotation schema obtained by InterProScan is shown in **Figure 4**, while long contigs without any explicit annotation related to phage structural proteins or functional ORFs are shown in Supplementary Figure S2.

**FIGURE 5:**
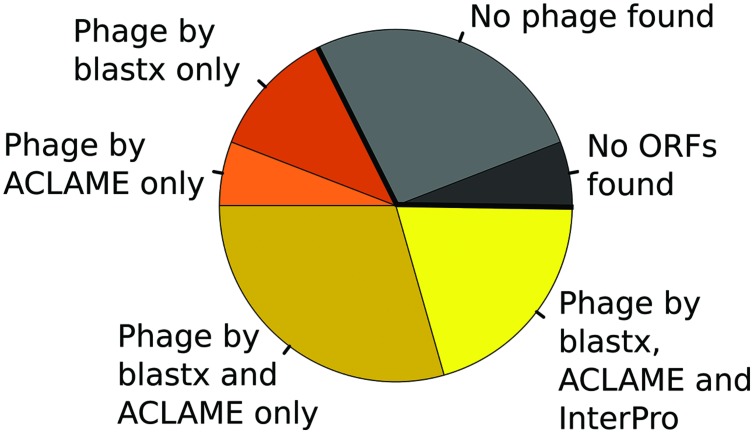
**Proportion of bacteriophage-related matches obtained by different annotation approaches.** The contigs were analyzed by “blastx” against the “nr” database, ACLAME and InterProScan. The majority of contigs had bacteriophage-related hits on at least one of the three approaches.

Moreover, the 34 long contigs were also analyzed by aligning to the NCBI “nr” database using the “blastx” program and to the ACLAME database of mobile genetic elements. Different annotation approaches applied to the long contigs helped to identify more contigs that could potentially contain bacteriophage-related sequences, shown in **Figure 5**. Ten contigs, which had been unannotated by InterPro, were finally related to bacteriophages by “blastx” and ACLAME. Two more contigs had bacteriophage hits on the ACLAME database only (but not “blastx”) and four more contigs matched potential phages on “blastx” (but not on ACLAME).

### Analysis of Unassembled Reads

Half (50.54%) of the unassembled sequences and contigs shorter than 1000 bp could not be assigned by “blast” to any organism on the “nr” database in our study (Supplementary Figure S3). The most frequent genera detected by “blast” on the NCBI “nr” database were *Bacteroides*, *Alistipes*, *Ruminococcus*, *Bifidobacterium*, *Roseburia*, *Eubacterium*, *Faecalibacterium*, and *Odoribacter* (Supplementary Figure S3). No human mitochondrial or ribosomal hits were found in the SSV-fraction. The alignment against the phiSITE treated database of viral genomes showed that short contigs and unassembled reads of the SSV-fraction contained sequences matching phages (110 matches, with an *e*-value < 0.00001; see Supplementary Figure S4).

## Discussion

Fluorescence activated cell sorting has been previously used for the sorting of cultivated phages and marine viruses (Chen et al., 2001; Brussaard, 2004; Allen et al., 2011). This work represents the first sorting of the viral particles coming from the human gut.

Fluorescence activated cell sorting proved to be very useful for additional purification of the fecal viromes previously filtrated through 0.2 μm pores. The size of non-viral genomes outnumbers viral genomes (3.5 kb to >1 Mb) 100s or 1000s of times. It means that just one bacterial or human cell accidentally present in the filtered sample may enormously contaminate the whole filtrate (Petrov et al., 2010). There are several reports on bacteria that are able to pass through filter pores (Hahn, 2004; Wang et al., 2007). The contamination by human ribosomal RNA or mitochondrial DNA is a frequent issue in human viromes; in some cases it can form up to 98% of all sequences (Willner et al., 2009). No human mitochondrial contamination was detected in our study, so FACS also represents an improvement in this issue.

The herein presented approach also has its strengths in the sequencing library preparation step. The amount of extracted viral DNA was below the minimal input amount required by the official library preparation protocol. We preferred to sequence the extracted viral DNA directly, avoiding the use of WGA and therefore DNA enrichment. We prepared the sequencing library using the modified protocol by Džunková et al. (2014), in which DNA losses during the library preparation are reduced. The resulting sequencing library contained a sufficient DNA amount for loading on the sequencing plate. It demonstrated that viral DNA can be successfully sequenced even if the extracted DNA is not enriched by WGA. Applying WGA to a viral sample can be very tricky, as the formation of chimeras during WGA could result in the misidentification and over-estimation of viral-like sequences (Lasken and Stockwell, 2007). Moreover, as the genome sizes of viruses are very variable (Edwards and Rohwer, 2005), some viral species can be over-amplified by WGA and some of them can be passed over (Kim et al., 2008).

The selection of viral particles with a similar size and DNA content by FACS seems to improve the assembly of viral genomes, because naturally lower number of species can be assembled more easily. The choice of the assembly algorithm also strongly influences the resulting length of viral contigs (Vázquez-Castellanos et al., 2014), which is what complicates comparisons of different studies. In the study by Minot et al. (2013), 56 Gbp was assembled into 478 long contigs. In comparison, 17.07 Mbp were assembled into 34 long contigs in our study, which represents a 3,280-fold improvement. The present study explores a small fraction of the entire fecal virome, but various different viral fractions can be separated by FACS during one sorting session. DNA from these fractions can be sequenced and assembled separately, thus a much larger diversity of a virome can be captured.

The contigs in our study mainly possessed genes typical for bacteriophages. This observation is in accordance with the results of many other studies that investigate healthy individuals (Reyes et al., 2010; Minot et al., 2012, 2013; Wagner et al., 2013), which emphasizes the importance of bacteriophages in the human gut. On the other hand, authors sequencing cDNA from fecal samples of healthy volunteers reported more eukaryotic viruses than bacteriophages, indicating that human viruses in feces might be recovered by RNA extraction (Zhang et al., 2006; Pérez-Brocal et al., 2013).

About half of the unassembled reads in our study could not be assigned to any organisms on the NCBI “nr” database by “blastn” algorithm, which is quite a common observation for the majority of virome studies. The reason for this is that the sequence databases are biased toward the most studied human viruses, and therefore the proportion of the sequences assigned to viruses is reported to be between 1.5 and 76% depending on sequence source (DNA/cDNA) and the data analysis method (Breitbart et al., 2008).

The bacterial matches are also very common in virome studies (Finkbeiner et al., 2008; Reyes et al., 2012) and can be explained by the fact that many phages are incorporated in the host genomes and are therefore present on the databases as parts of microbial genomes (Ghosh et al., 2008; Stern et al., 2012). The bacterial species matched by “blastn” approach in our study were the same as the species reported by Minot et al. (2013) and Ogilvie et al. (2013).

## Conclusion

Our results indicate that a filtered viral sample contains particles that can be further divided by FACS into fractions according to their size and fluorescence. FACS coupled with the alternative sequencing library preparation protocol omitting WGA helped to solve the difficulties reported in many virome sequencing projects. Our approach reduces the sequencing force needed for assembling viral sequences into larger contigs and avoids contamination.

## Author contributions

MD, GD, and AM conceived and designed the experiments. MD performed the experiments. MD and GD analyzed the data. MD, GD, and AM wrote the paper.

## Conflict of Interest Statement

The authors declare that the research was conducted in the absence of any commercial or financial relationships that could be construed as a potential conflict of interest.
