# Supplementary material for: Direct sequencing of human gut virome fractions obtained by flow cytometry
Source: Front Microbiol. 2015 Sep 8;6:955. doi: 10.3389/fmicb.2015.00955 (PMC4568480; doi:10.3389/fmicb.2015.00955)

Supplementary Information

Title: Direct sequencing of human gut virome fractions obtained by flow cytometry

Authors: Mária Džunková, Giussepe D'Auria, Andrés Moya

Supplementary Information Figure S1

Laboratory workflow of fecal virome purification and staining for flow cytometry

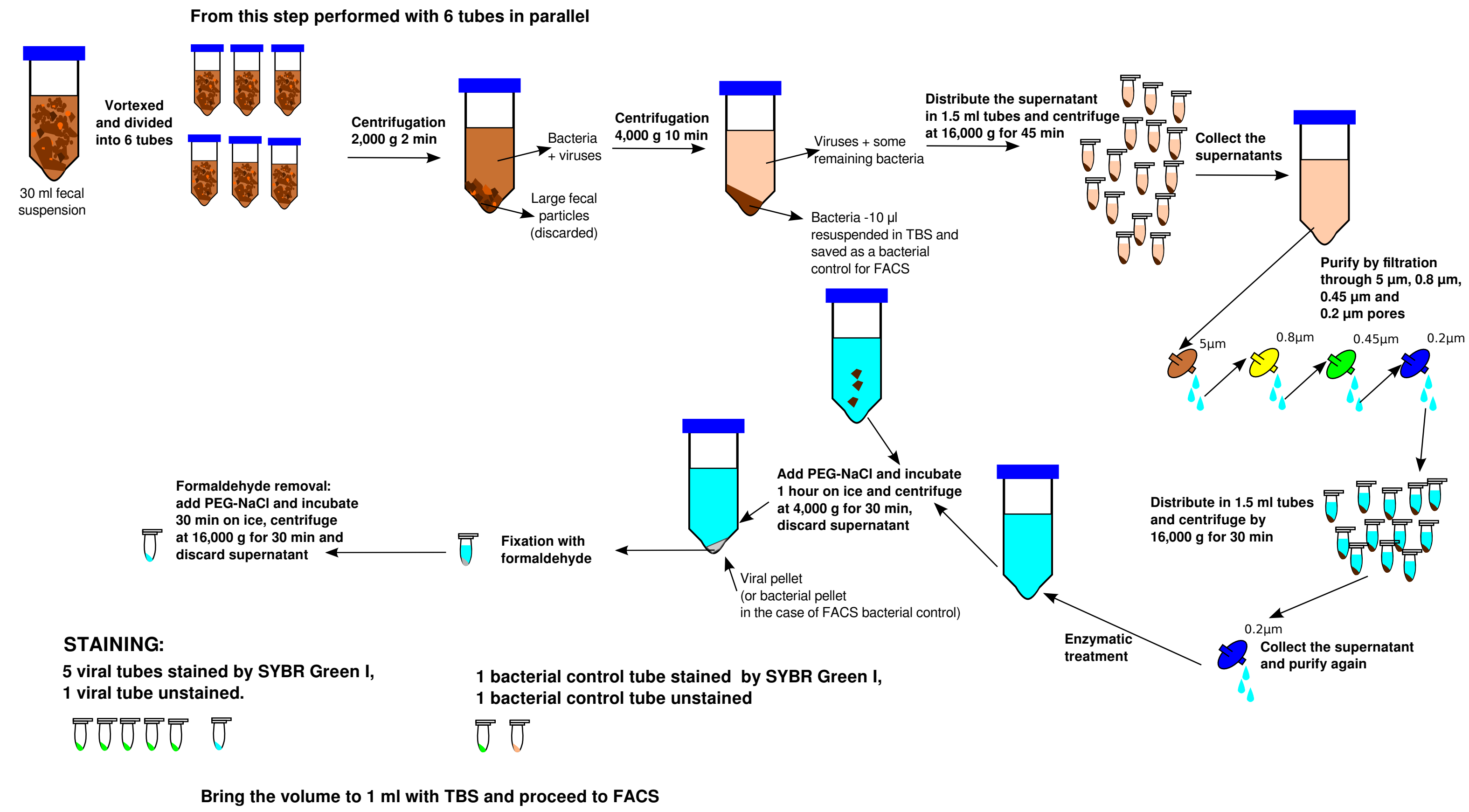

Supplementary Information Figure S2

Visualization of contigs that had not explicit matches to phage related proteins by InterProScan. Annotation by all searching tools included in this database is shown, as well as Glimmer detected ORFs. The visualization of contigs that had explicit matches to phage related proteins is shown in the main article text in Figure 4.

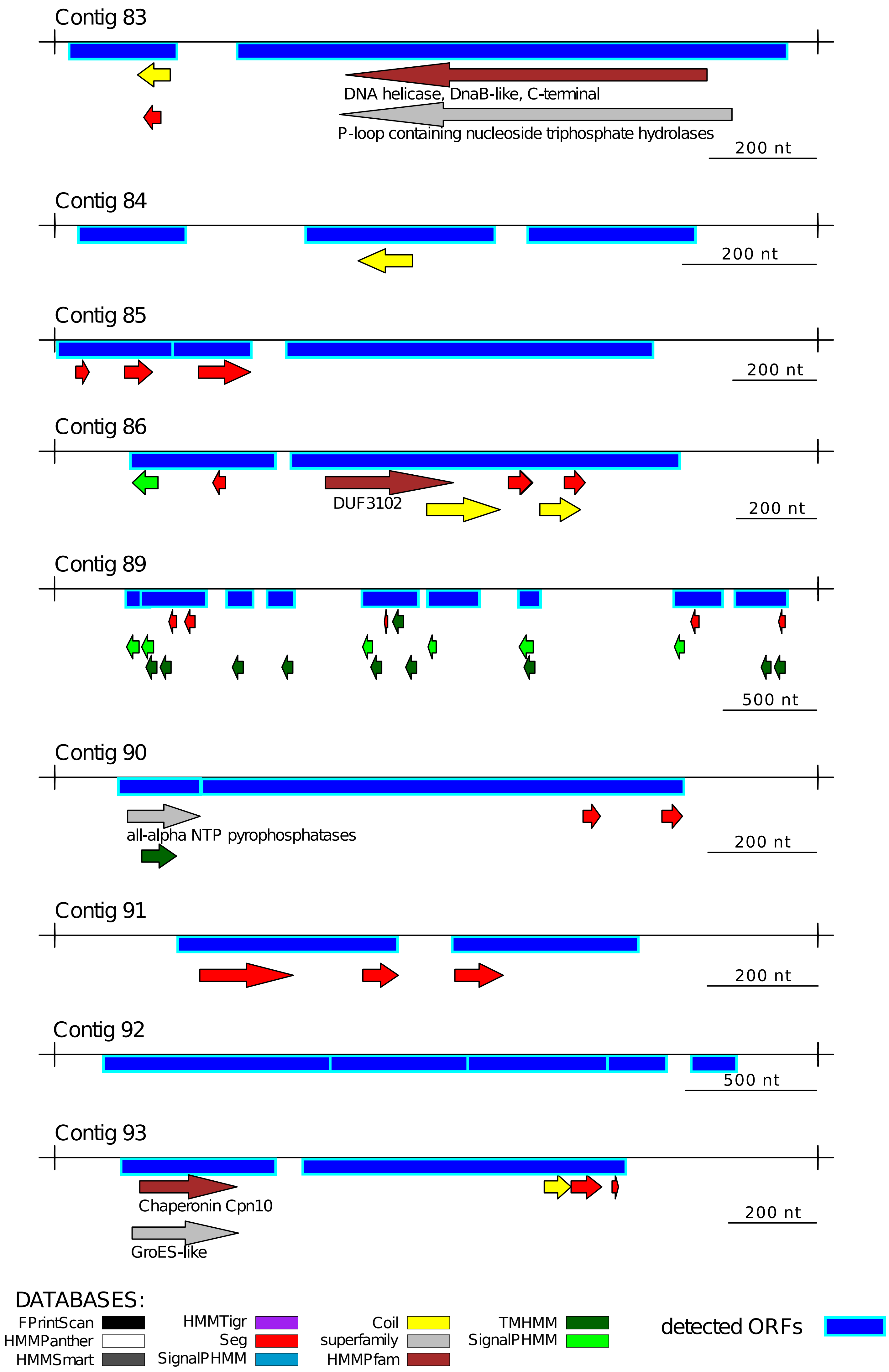
Contig 83
DNA helicase, DnaB-like, C-terminal
P-loop containing nucleoside triphosphate hydrolases
200 nt
Contig 84
200 nt
Contig 85
200 nt
Contig 86
DUF3102
200 nt
Contig 89
500 nt
Contig 90
all-alpha NTP pyrophosphatases
200 nt
Contig 91
200 nt
Contig 92
500 nt
Contig 93
Chaperonin Cpn10
GroES-like
200 nt
DATABASES:
FPrintScan
HMMPanther
HMMSmart
HMMTigr
Seg
SignalPHMM
Coil
superfamily
HMMPfam
TMHMM
SignalPHMM
detected ORFs

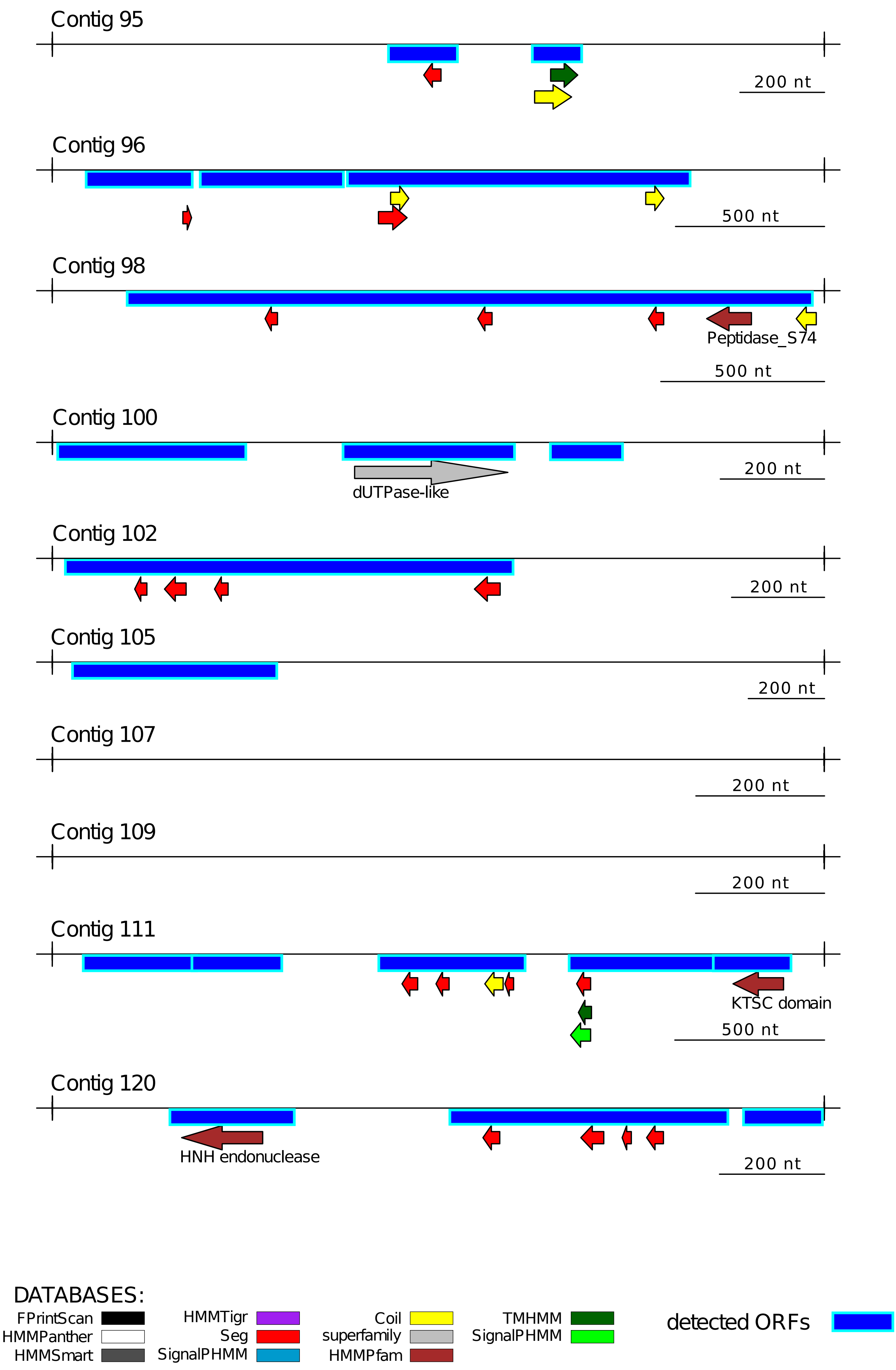
Contig 95
200 nt
Contig 96
500 nt
Contig 98
Peptidase_S74
500 nt
Contig 100
200 nt
dUTPase-like
Contig 102
200 nt
Contig 105
200 nt
Contig 107
200 nt
Contig 109
200 nt
Contig 111
KTSC domain
500 nt
Contig 120
HNH endonuclease
200 nt
DATABASES:
FPrintScan
HMMPanther
HMMSmart
HMMTigr
Seg
SignalPHMM
Coil
superfamily
HMMPfam
TMHMM
SignalPHMM
detected ORFs

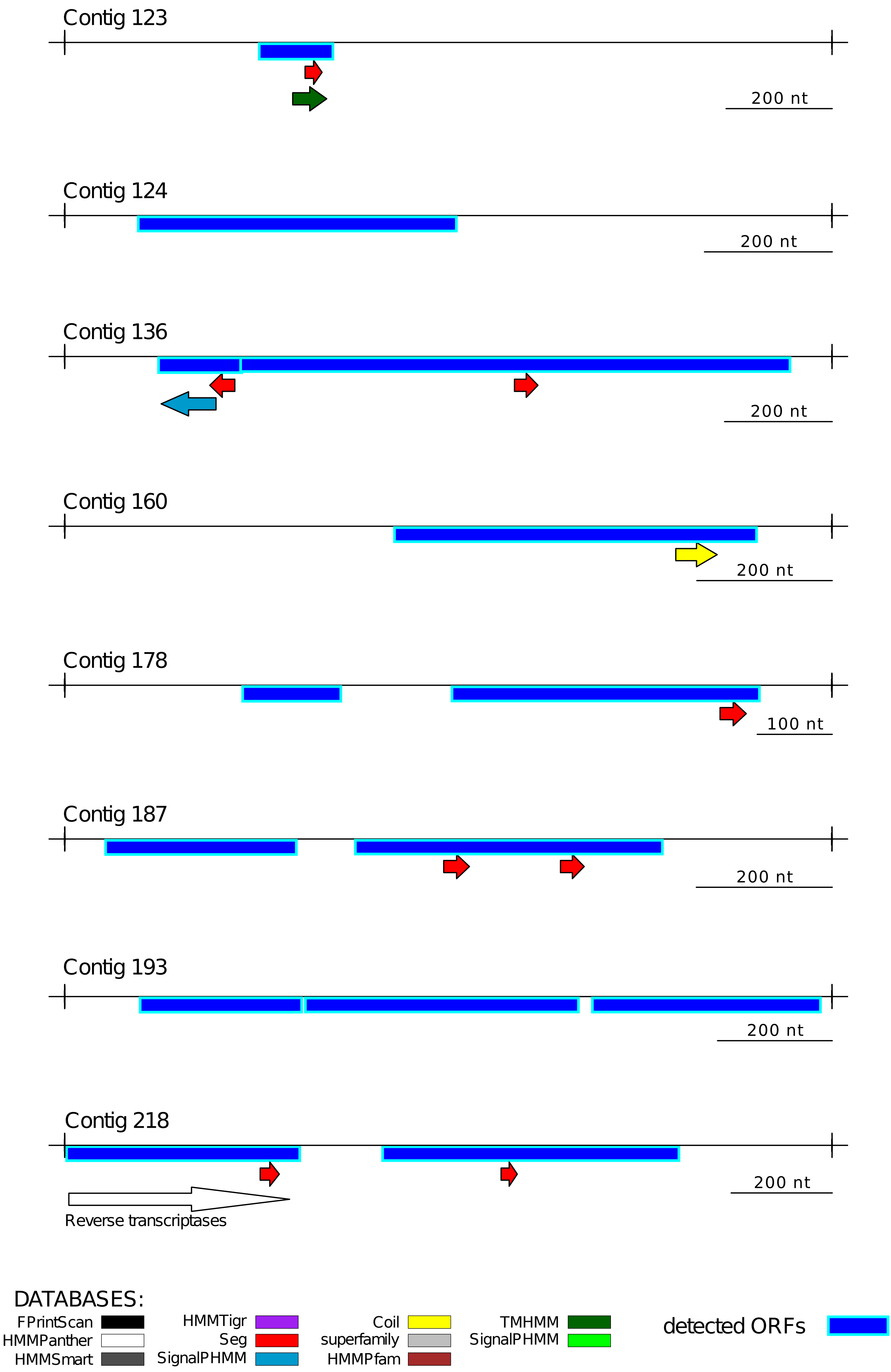
Contig 123
200 nt
Contig 124
200 nt
Contig 136
200 nt
Contig 160
200 nt
Contig 178
100 nt
Contig 187
200 nt
Contig 193
200 nt
Contig 218
200 nt
Reverse transcriptases
DATABASES:
FPrintScan
HMMPanther
HMMSmart
HMMTigr
Seg
SignalPHMM
Coil
superfamily
HMMPfam
TMHMM
SignalPHMM
detected ORFs

Supplementary Information Figure 3

Barplot showing composition of species in unassembled sequences and contigs shorter than 1000 bp detected by “blastn” approach. The upper pie-charts show proportion of reads assigned to “nr” database.

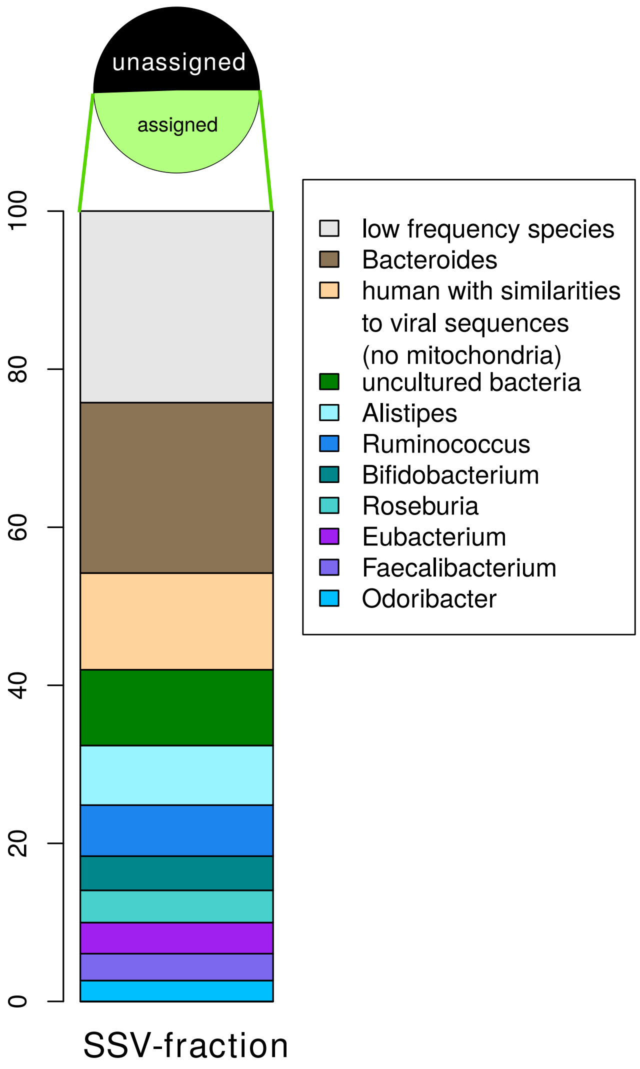

Supplementary Information Figure S4

Best matches to phiSITE database of all unassembled sequences and contigs shorter than 1000 bp of SSV-fraction. The graphics shows the number of matches to the hosts names.

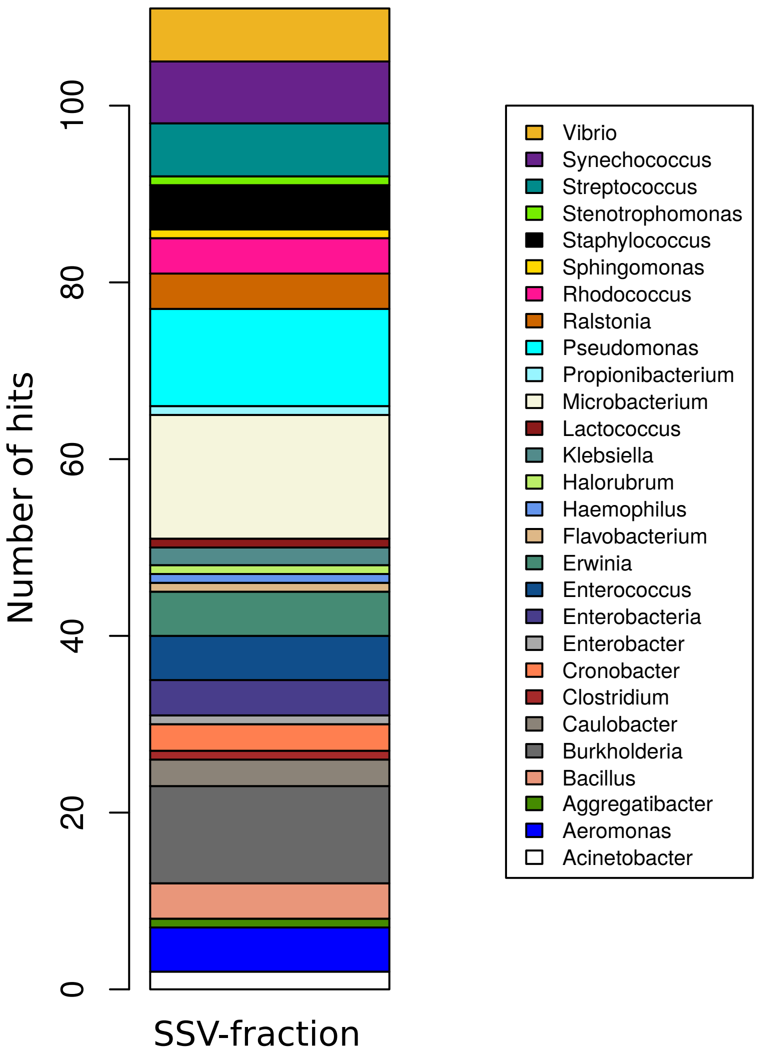

Supplement: Supplementary file 1 [file Image_1.PDF]
